# Paints and Dyes

**Published:** 1889-12

**Authors:** 


### PAINTS AND DYES.

The cochineal insects furnish a great many of the very fine colors. Among them are the gorgeous carmine, the crimson, scarlet carmine, and purple lakes. The cuttlefish gives the sepia. It is the inky fluid which the fish discharges in order to render the water opaque when attacked. Indian yellow comes from the camel. Ivory chips produce the ivory black and bone black. The exquisite Prussian blue is made by fusing horses' hoofs and other refuse animal matter with impure potassium carbonate. This color was discovered accidentally. Various lakes are derived from roots, barks, and gums. Blue-black comes from the charcoal of the vine stalk. Lamp black is soot from certain resinous substances. Turkey red is mud from the madder plant, which grows in Hindostan. The yellow sap of a tree of Siam produces gamboge; the natives catch the sap in cocoanut shells. Raw sienna is the natural earth from the neighborhood of Sienna, Italy. Raw umber is also an earth found near Umbria and burnt. India ink is made from burnt camphor. The Chinese are the only manufacturers of this ink, and they will not reveal the secret of its manufacture. Mastic is made from the gum of the mastic tree, which grows in the Grecian Archipelago. Bistre is the soot of wood ashes. Very little real ultramarine is found in the market. It is obtained from the precious lapis-lazuli, and commands a fabulous price. Chinese white is zinc, scarlet is iodide of mercury, and native vermilion is from the quicksilver ore called cinnebar.

### COMMERCIAL MARRIAGES.

Commercial marriages are becoming conspicuously frequent of late between impecunious European "noblemen" (?) and foolish American heiresses. Not long since, "Prince Hatzfeld," a bankrupt young German aristocrat, became the husband of Miss Huntington, of New York. It has been settled that he is to share, with his wife, the income of the three million dollars which she owns, and the prospect is held out that the father-in-law will eventually pay the Prince's debts, which amount to half a million dollars in lieu of which she shares his empty title and becomes a princess. At the same time Paris has been agog over the prospective marriage between "Prince Murat," a French nobleman, and Miss Caldwell, the New Orleans heiress, who made herself famous by endowing the new Catholic university at Washington. At latest accounts this match is declared off, though the bridal trosseau was ready at Worth's, because they could not agree in settling financial matters. These mesalliances are a sickly sign of the spirit of our times. That bankrupt scions of titled families should be anxious to wed American wealth, is not so strange, but that beautiful, "accomplished" millionairesses in this free country should be willing to bestow their money, if not their affections, upon these European scapegraces, most of whom are moral lepers, for the sake of having a *title*, is supremely disgusting. For that is what it is : worthless titles are traded off for wealth in the marital market. The American women are, in such cases, by far the greatest dupes. European titles, however high sounding are, in this late day as empty as a child's whistle.—*Evangelical Messenger.*

